# Biodiversity of network modules drives ecosystem functioning in biochar-amended paddy soil

**DOI:** 10.3389/fmicb.2024.1341251

**Published:** 2024-01-24

**Authors:** Yu Xiao, Guixiang Zhou, Xiuwen Qiu, Fangming Liu, Lin Chen, Jiabao Zhang

**Affiliations:** ^1^State Key Laboratory of Soil and Sustainable Agriculture, Institute of Soil Science, Chinese Academy of Sciences, Nanjing, China; ^2^University of Chinese Academy of Sciences, Beijing, China; ^3^College of Landscape Architecture, Jiangsu Vocational College of Agriculture and Forestry, Jurong, China

**Keywords:** soil microbial biodiversity, biochar amendment, ecosystem functioning, core modules, protists

## Abstract

**Introduction:**

Soil microbes are central in governing soil multifunctionality and driving ecological processes. Despite biochar application has been reported to enhance soil biodiversity, its impacts on soil multifunctionality and the relationships between soil taxonomic biodiversity and ecosystem functioning remain controversial in paddy soil.

**Methods:**

Herein, we characterized the biodiversity information on soil communities, including bacteria, fungi, protists, and nematodes, and tested their effects on twelve ecosystem metrics (including functions related to enzyme activities, nutrient provisioning, and element cycling) in biochar-amended paddy soil.

**Results:**

The biochar amendment augmented soil multifunctionality by 20.1 and 35.7% in the early stage, while the effects were diminished in the late stage. Moreover, the soil microbial diversity and core modules were significantly correlated with soil multifunctionality.

**Discussion:**

Our analysis revealed that not just soil microbial diversity, but specifically the biodiversity within the identified microbial modules, had a more pronounced impact on ecosystem functions. These modules, comprising diverse microbial taxa, especially protists, played key roles in driving ecosystem functioning in biochar-amended paddy soils. This highlights the importance of understanding the structure and interactions within microbial communities to fully comprehend the impact of biochar on soil ecosystem functioning in the agricultural ecosystem.

## 1 Introduction

Soils, a main repository of terrestrial biodiversity, harbors one-quarter of all species on Earth, including millions of species and billions of organisms (Bardgett and Van Der Putten, 2014; Jansson and Hofmockel, 2020; Guerra et al., 2021; Coban et al., 2022). Soil microorganisms are highly diverse and complex, spanning from minute bacteria and fungi to more sizable organisms, such as protists and nematodes (Bardgett and Van Der Putten, 2014; Guerra et al., 2021). As a large fraction of soil biome, soil microbiome is undeniably a crucial component in both natural and anthropogenic ecosystems, influencing soil pH, structure, and fertility. Additionally, they can also impact soil water availability by modifying soil hydraulic conductivity and hydrophobicity (Fierer, 2017). Soil microbiome is inseparably linked to soil health, serving as a critical driver for One Health initiatives aimed at promoting ecosystem health (Lehmann et al., 2020).

Soil microorganisms govern a wide variety of fundamental functions that underpin essential ecosystem services (Fierer, 2017). The microbes are involved in ecosystem biogeochemical processes on Earth, driving macronutrients, micronutrients, and other elements cycling with direct feedback effects on soil ecosystem functioning (Fierer, 2017; Jansson and Hofmockel, 2020). There is a number of evidence that soil biodiversity can significantly impact the soil multifunctionality in diverse ecosystems (Nielsen et al., 2011; Delgado-Baquerizo et al., 2016, 2020; Chen et al., 2020). The positive relationships between biodiversity and multifunctionality were observed in terrestrial ecosystems and urban greenspaces (Delgado-Baquerizo et al., 2016; Fan et al., 2023). The multifunctionality also exhibited a strong linear correlation with soil biodiversity in model grassland (Wagg et al., 2014). Yet the evidence for this conclusion primarily stems from experiments testing the impact of multidiversity or solely considering the effects of low-trophic-level soil microorganisms on multifunctionality (Wagg et al., 2019; Chen et al., 2020), despite the fact that the ecosystem functions are dominated by certain trophic organisms. While extensive understanding exists regarding the regulation of ecosystem multifunctionality by soil biodiversity, the identification of specific biological populations crucial to sustaining these ecosystem functions remains an unresolved challenge.

Biochar has received a great deal of attention as an effective soil amendment for enhancing soil fertility and promoting plant growth. With its ability to improve soil structure, nutrient cycling, and fertility, biochar has emerged as a promising sustainable method of soil improvement in agriculture (Zhou et al., 2019). In the terrestrial ecosystem, a broad range of biogeochemical processes, including carbon sequestration, soil respiration, ammonification and nitrification, and nutrient transport and transformation, are affected by biochar application (Liang et al., 2017; Zheng et al., 2018; Dong et al., 2022). Over the past few decades, studies have reported that biochar can enhance soil microbial functional activities and optimize community structure, thereby augmenting soil biological properties (Li et al., 2018). The highly porous structure and various functional groups of biochar provide habitats for soil microorganisms to colonize and further stimulate microbial activities (Liu et al., 2018). Biochar amendment can also significantly modify soil physicochemical properties, such as soil pH and organic matter content, creating a more suitable environment for microbial growth, development, and proliferation (Palansooriya et al., 2019). However, the role of soil microbes on ecosystem functioning in paddy soil amended with biochar remains comparatively unexplored. Identifying mechanisms by which biochar modulates the diversity-multifunctionality relationships within the soil ecosystem is essential to comprehending how biochar application affects the paddy soil ecosystem.

Herein, this work was to (1) investigate the effects of biochar on soil multifunctionality, and (2) evaluate the relationship between interactions among different microorganisms and their functions. We characterized the biodiversity information on bacteria, fungi, protists, and nematodes in soil samples collected from a biochar-amended paddy soil. Twelve ecosystem metrics from the enzyme level to nutrient provisioning and their simultaneous provision (multifunctionality) were adopted. The soil network among the multitrophic members of the soil microbiome was visualized using co-occurrence networks.

## 2 Materials and methods

### 2.1 Experimental design and soil sampling

The pot experiment was carried out in Jiujiang City (29°68′N, 115°98′E), Jiangxi Province, South-Eastern China, which features a subtropical warm and humid monsoon climate, exhibiting four distinct seasons. The paddy soil (classified as Stagnic Anthrosols) was collected from local farmland in Jiujiang before the 2020 rice planting season. There are four treatments with three replicates. Maize straw biochar, which pyrolyzed at 400°C, was used with the dosage of 2.5 g kg^–1^ (B1), 5.0 g kg^–1^ (B2), and 10.0 g kg^–1^ (B3) in biochar treatments, respectively. Pots (20 cm × 20 cm × 25 cm) without biochar amendment were treated as control (CK). In the Spring of 2020, rice seedlings (Taifengyou3301) were planted in plastic pots with 10.0 kg soil. The total number of pots was 4 biochar levels × 2 sampling times (heading stage and maturation stage of rice) × 3 replicates = 24 pots. The pot experiment was initiated in May 2020, with sample collection during the heading stage (*n* = 36) conducted in August 2020, followed by the maturity stage (*n* = 36) sampling in October 2020. The experimental duration lasted for 4 months. During the maturity stage sampling, the average seedling height measured 90 cm. After sampling, the soil was separated into three aggregate classes: >2000 μm (large macroaggregate), 250–2000 μm (small macroaggregate), and <250 μm (microaggregate) at each sampling time following the previous study (Elliott, 1986).

### 2.2 Ecosystem functions

In the present study, we quantified 12 metrics related to enzyme activities, nutrient provisioning, and element cycling: soil organic carbon (SOC), dissolved organic C (DOC), total N (TN), total P (TP), ammonium (NH_4_^+^), nitrate (NO_3_^–^), saccharase, β-glucosidase, urease, phosphatase, available S (AS) and available Fe. The contents of SOC, total N, total P, ammonium, and nitrate were measured using standard soil testing procedures (Hu et al., 2021). The concentrations of DOC, available S, and available Fe were detected following the previous approach (Jiao et al., 2022). The extracellular enzyme activities were determined using the model substrates methods as described previously (Yao et al., 2019; Han et al., 2021). Ecosystem multifunctionality measures reflect the ability of an ecosystem to supply multiple functions or services simultaneously. To deliver a quantitative multifunctionality index for each sample, the 12 metrics measured were normalized and standardized using the Z-score transformation. After standardizing, the ecosystem functions were averaged to calculate the multifunctionality index (Delgado-Baquerizo et al., 2016).

### 2.3 Soil biodiversity analysis

Soil microbial DNA was extracted from fresh soil in three aggregate levels by using the Fast DNA SPIN Kit (MP Biomedicals, Santa Ana, CA, USA) according to the manufacturer’s instructions. The diversity and composition of bacteria, fungi, protists, and nematodes were analyzed using sequencing (Illumina HiSeq-PE250 platform). Bacterial 16S rRNA was amplified the V4-V5 region with the primer pairs: 5′-GTGCCAGCMGCCGCGGTAA-3′ and 5′-CCGTCAATTCMTTTRAGTTT-3′. Eukaryota 18S rRNA was amplified using the paired primers: 5′-GGTGGTGCATGGCC GTTCTTAGTT-3′ and 5′-TACAAAGGGCAGGGACGTAAT-3′ (Fan et al., 2021). Bacterial and eukaryotic sequences have been submitted to the NCBI Sequence Read Archive (SRA) with the accession number PRJNA984952.

Raw sequences were processed using the QIIME pipeline and then clustered into operational taxonomic units (OTUs) at a 97% similarity threshold after removing the low-quality sequence. The SILVA database was used to classify bacteria and nematodes, while the UNITE database and the Protist Ribosomal Reference (PR2) database were employed for the classification of fungi and protists, respectively, with all taxonomic assignments adhering to a 97% similarity threshold. The even sampling depths for each group were 12049 for bacteria, 2006 for fungi, 7524 for protists, and 119 for nematodes. Soil biodiversity was characterized as richness (i.e., number of soil phylotypes) and Shannon diversity. The multidiversity index was calculated by averaging the standardized values (0–1) of richness of all types of organisms: soil bacteria, fungi, protists, and nematodes (Wang et al., 2019).

### 2.4 Statistical analyses

We chose the abundant (top 10% of all identified bacteria and eukaryotes) phylotypes to construct the co-occurrence networks. All pairwise Spearman correlations were calculated in the package “WGCNA” in R and the correlation threshold was above 0.65 and *P*-value < 0.01. Additionally, we employed the analysis of variance (ANOVA) to eliminate the variance. The data for network analysis is derived from the OTU data of all 72 samples, obtained at various aggregation levels of microbial OTUs. The networks were visualized using Gephi software. The modules were identified from soil microbial groups that were closely interacting with each other. Structural equation models (SEMs) were used to assess the direct and indirect effects of soil pH, biochar, soil biodiversity, and modules on multifunctionality. The data used for the structural equation model (SEM) is based on 72 samples. For bacteria, fungi, protists, and nematode diversity data, we utilized richness data. Regarding module data, we performed principal component analysis (PCA) on the relative abundances of modules 1, 2, 3, and 4 and subsequently extracted the data from the first principal component (PCA1). SEM was conducted in IBM SPSS Amos 21. Linear regressions (based on Spearman correlations) were performed to establish the relationships between soil biodiversity, multidiversity, and multifunctionality. Heatmaps were conducted to estimate the Spearman correlations between biodiversity, modules, and single functions. We also evaluated the role of main protists and nematodes in multifunctionality by linear regressions.

## 3 Results

### 3.1 Effect of biochar addition on soil multifunctionality

**FIGURE 1:**
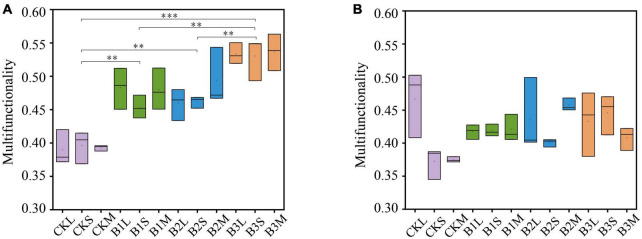
The multifunctionality in different treatments. **(A)** Early stage of rice growing, **(B)** late stage of rice growing. CK, control, and without additive; B1, with the addition of 2.5 g kg^–1^ biochar; B2, with the addition of 5 g kg^–1^ biochar; B3, with the addition of 10 g kg^–1^ biochar; L: large macroaggregates, S, small macroaggregates, M, microaggregates. *P-*values were indicated by asterisks: *P < 0.05, **P < 0.01, ***P < 0.001, and ****P < 0.0001.

We examined the influence of biochar addition on soil ecosystem multifunctionality at different aggregate sizes. The results suggested that the addition of biochar significantly enhanced the soil multifunctionality compared to the control treatment in the early stage (*P* < 0.01) (Figure 1A). Moreover, soil multifunctionality increased along with the increasing doses of biochar application in the early stage. For example, the soil ecosystem multifunctionality in B1 and B3 increased by 20.1 and 35.7%, respectively, compared to that in CK (Supplementary Figure 1A). Nevertheless, the impacts of biochar amendment on soil multifunctionality were inconsistent at different stages. The biochar amendment had a significantly positive effect (*P* < 0.01) on soil multifunctionality in the early stage, but the effect was diminished in the late stag (*P* > 0.05) (Figures 1A, B and Supplementary Figure 1). These results suggested that the biochar amendment exhibited a stronger positive association with soil multifunctionality in the early stage, and such an effect was enhanced by the increase of biochar application. At the late stage, however, biochar application exhibited weaker or no relationships with soil ecosystem multifunctionality compared to the control treatment. At the aggregate level, no significant correlations were observed for biochar amendment on multifunctionality (Figure 1A). Regarding aggregate size, in the early stage, the highest biodiversity is observed in the microaggregates (Supplementary Figure 2), with a gradual decrease noted in larger aggregate sizes. The large aggregates exhibit the lowest biodiversity during this stage. In the late stage, there is a notable increase in biodiversity for small aggregates, which exhibit the highest biodiversity, followed by microaggregates, with large aggregates displaying the lowest biodiversity among the three categories (Supplementary Figure 2).

### 3.2 Relationship between microbial diversity and soil multifunctionality

**FIGURE 2:**
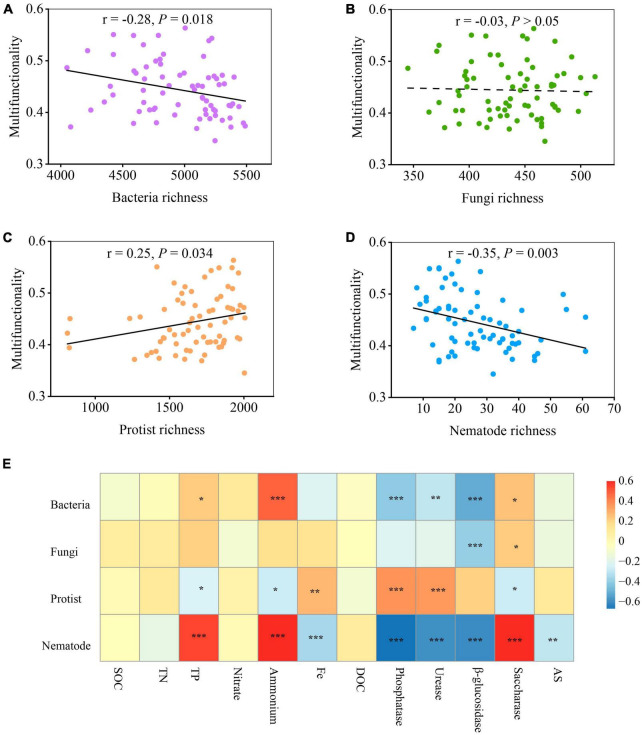
The relationship between multifunctionality and biodiversity of organisms with biochar addition. **(A–D)** The linear relationships between multifunctionality and the biodiversity of selected groups of soil organisms (averaged standardized between 0 and 1). Statistical analysis was performed using ordinary least squares linear regressions; *P-*values were indicated by asterisks: *P < 0.05, **P < 0.01, and ***P < 0.001. **(E)** Significant correlations (Spearman; ρ < 0.05) between the diversity of single groups of organisms and 12 single ecosystem metrics (including functions related to enzyme activities, nutrient provisioning, and element cycling). SOC, soil organic carbon; TN, total nitrogen; TP, total phosphorus; DOC, dissolved organic carbon; AS, available sulfur.

The relationship between microbial diversity and soil multifunctionality was explored using the standardized average of twelve variables: SOC, TN, TP, nitrate, ammonium, Fe, DOC, phosphatase, urease, β-glucosidase, saccharase, and AS (see section “2 Materials and methods”). The results indicated that the diversity of soil organisms shifted the soil ecosystem multifunctionality (Figures 2A–D and Supplementary Figure 3). The least-squares regression models revealed a negative linear correlation between soil microbial richness and soil multifunctionality for both bacteria (*r* = −0.28, *P* = 0.018) and nematodes (*r* = −0.35, *P* = 0.003) (Figures 2A, D). In contrast, the richness of protists was positively related to soil ecosystem multifunctionality along with the soil diversity gradient (*r* = 0.25, *P* < 0.05) (Figure 2C). However, the fungal richness showed weaker or no multifunctional relationships than the richness of bacteria, protists, and nematodes (Figure 2B). Interestingly, this relationship was found to be different when considering the Shannon index (Supplementary Figure 3). Unlike in Figure 2B, the fungal Shannon diversity significantly decreased soil ecosystem multifunctionality (*P* = 0.01), but weaker or no significant associations between the Shannon diversity of bacteria and nematodes and soil multifunctionality were observed (*P* > 0.05) (Supplementary Figure 3). Notably, the relationship between the diversity of protists and soil multifunctionality was maintained when using the Shannon index (Supplementary Figure 3). The diversity of protists was the only exception presenting a significantly positive effect when both richness and Shannon index were employed as an indicator to evaluate soil microbial diversity (Figure 2C and Supplementary Figure 3).

By identifying the correlation between the diversity of single groups of organisms and single ecosystem functions, it was observed that various soil organisms had distinct impacts on the different aspects of ecosystem functions and services (enzyme activities, nutrient provisioning, and element cycling) (Lefcheck et al., 2015; Jiao et al., 2022). Among them, soil organic carbon (SOC), dissolved organic C (DOC), total N (TN), total P (TP), ammonium (NH_4_^+^), and nitrate (NO_3_^–^) represent nutrient provisioning. Saccharase, β-glucosidase, urease, and phosphatase are indicative of enzyme activities, while available S (AS) and available Fe are associated with element cycling. Activities of P-, N-, and C- cycle enzymes, including phosphatase, urease, β-glucosidase, and saccharase, showed significant correlations with different soil microbes (bacteria, fungi, protists, and nematodes) (Figure 2E). Among the enzymes assessed, the activities of phosphatase, urease, and β-glucosidase were negatively related to the diversity of bacteria and nematodes, while the activities of phosphatase and urease positively correlated with the diversity of protists (Figure 2E). In particular, saccharase was the only enzyme displaying positive association with most of the microorganisms, especially on nematodes (*P* < 0.001) (Figure 2E). In addition, the accumulation of NH_4_^+^ and TP was positively associated with the diversity of bacteria and nematodes but were not positively related to that of fungi and protists. No correlation, however, was found between some single ecosystem functions (including the accumulation of TN, SOC, NO_3_^–^, and DOC) and the diversity of four soil groups. Fe-element cycling and AS in soil were negatively associated with nematodes but positively with protists (Figure 2E).

To explore whether the biochar addition and multidiversity showed a significant correlation with multifunctionality, we further averaged the standardized scores (z scores) of all ecosystem functions to obtain a single index of soil ecosystem multifunctionality (Supplementary Figure 4). Similarly, the multidiversity index reflects the overall community compositional changes in concert with changes in soil microbial biodiversity. The biochar amendment exhibited highly significant positive effects on multifunctionality (*P* < 0.001), whereas the changes in ecosystem multifunctionality had negative associations with the average of the indicators of soil biodiversity (*P* < 0.05) (Supplementary Figure 4). These results suggested that the biochar amendment and changes in soil biodiversity significantly impact ecosystem multifunctionality in paddy soil.

### 3.3 Links between main phylotypes, modules, and ecosystem functions

**FIGURE 3:**
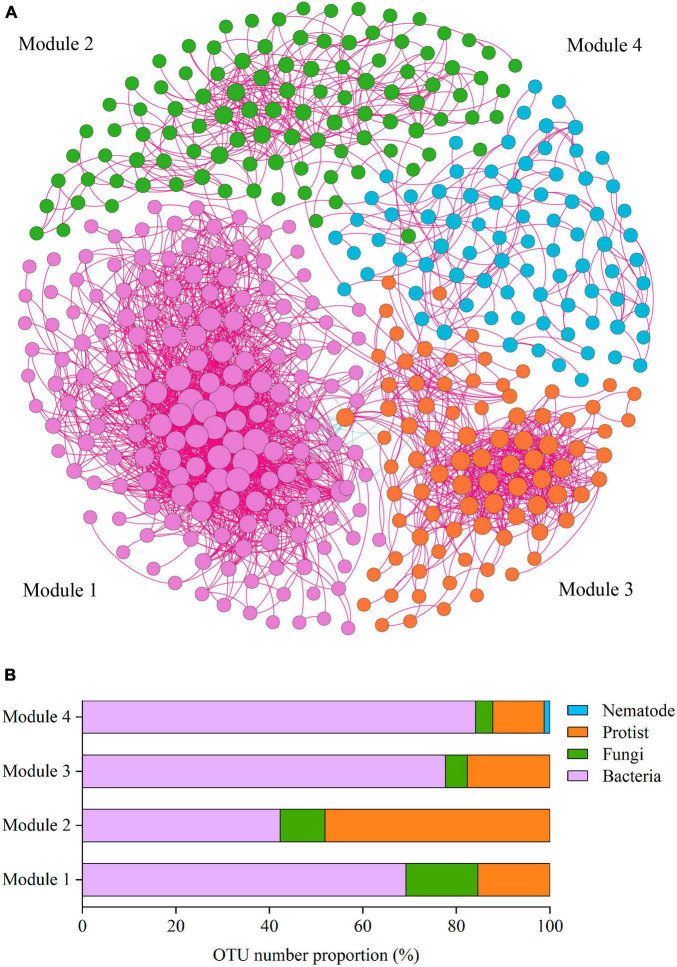
The main modules in the network and the OTU number proportion in the modules. **(A)** Co-occurrence network of soil microbial OTUs. Co-occurrence networks visualizing significant correlations (Spearman’s correlation coefficient >0.65) between OTUs in the communities of four kinds of soil microbial taxa (bacteria, fungi, protists, and nematodes). Four modules in microbial networks are shown in different colors. The size of each node accounts for the degree of OTUs, representing the connectedness among OTUs. The connecting lines (edges) among those nodes represent the interactions between soil organisms. The red and blue edges show positive and negative interactions, respectively. **(B)** OTUs number proportion (%) of the soil organismal phylotypes in Modules 1–4 in the network.

To reveal how soil phylotypes of different modules impact the soil ecosystem multifunctionality, the soil co-occurrence networks were constructed for four soil groups (bacteria, fungi, protists, and nematodes) (Figure 3). The integrated co-occurrence networks were composed of 440 nodes and 2582 edges. As shown in Figure 3A, positive correlations dominated the co-occurrence networks. The proportion of positive associations occupied 99.3% for the total edges of the network. Based on the co-occurrence networks, the multitrophic network was clustered into four modules (subunits with highly inter-connected nodes) (Figure 3A). Co-occurrence network analysis showed that a higher number of nodes and edges among nodes was observed in Module 1, indicating that Module 1 was more closely and complicatedly with higher connectedness than other modules (Figure 3A). We further measured the OTUs proportion of dominant taxa in Modules 1–4 and found that protist OTUs accounted for a considerable proportion of all soil microbial organisms within modules, with the highest relative abundance reaching 48.08% (Figure 3B).

**FIGURE 4:**
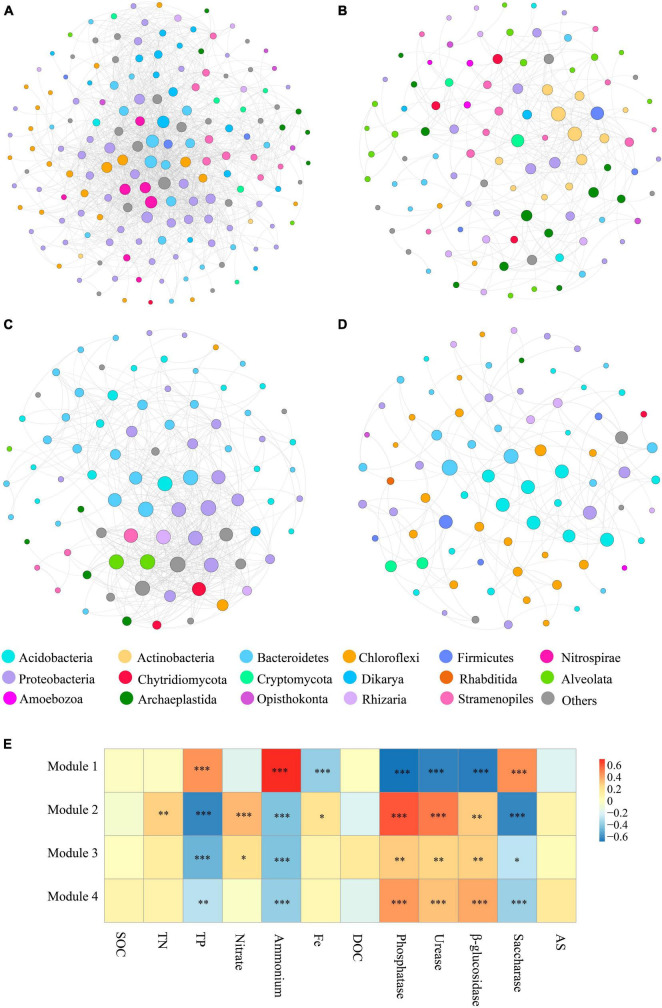
The main modules and the correlation between modules and physicochemical properties. **(A–D)** Taxonomic composition of modules. The different soil organisms are shown in different colors. The size of each node accounts for the degree of OTUs, representing the connectedness among OTUs. The connecting lines among those nodes represent the interactions between soil organisms. **(E)** Significant correlations (ρ < 0.05) between the modules and single ecosystem functions. *P-*values were indicated by asterisks: *P < 0.05, **P < 0.01, ***P < 0.001, and ****P < 0.0001.

To characterize microbial interactions within each module, we next structured the microbial networks of four ecological assemblies based on the co-occurrence network of total microbial OTUs. Consistent with the results presented in Figure 3A, the network of Module 1 contained a higher number of significantly co-occurring OTUs (nodes = 169) than the other networks. The network complexity within Module 1, as indicated by the number of edges (1557), was also higher than in other modules. The keystone taxa, represented by nodes with both high degree and low betweenness centrality values in the networks, differed among various modules (Figures 4A–D). The keystone species at the phylum level, found in Module 1, were Bacteroidetes and Nitrospirae, and Dikarya, which belong to bacteria and fungi, respectively (Figure 4A). Within Module 2, these were Actinobacteria, Firmicutes, Cryptomycota, and Archaeplastida, which belong to bacteria, fungi, and protists, respectively (Figure 4B). For Module 3, Proteobacteria, Bacteroidetes, and Alveolata which belong to bacteria and protists, respectively, were found to be the keystone phyla (Figure 4C). And the keystone phyla with Module 4 were Bacteroidetes, Acidobacteria, Firmicutes, and Proteobacteria (all belonging to bacteria) (Figure 4D).

It was found that protists displayed a high connectedness in the networks. Likewise, at the genus level, the affiliation of keystone taxa also varied significantly among the modules. Major keystone protistan species within Module 1 included members of the phylum Stramenopiles, comprising the genus *Navicula*, *Sellaphora*, and *Nitzschia*; the phylum Alveolata, consisting of the genus *Furgasonia*, and the phylum Opisthokonta, comprising the genus *Chaetonotida*, *Monogononta*, *Gastropoda*, and *Crustacea* (Figure 4A). Within Module 2, the majority of keystone protistan taxa were the phylum Stramenopiles, including the genus *Stauroneis* and *Pinnularia*; the phylum Alveolata, including the genus *Stylonychia*, *Cyrtolophosidida*, and *Didinium*, and the phylum Opisthokonta, comprising the genus *Monogononta* (Figure 4B). Most keystone protistan genera within Module 3 were composed of *Neidium* and *Cyrtolophosidida*, while within Module 4, they were *Rhabditida* and *Chromadorea* (Figures 4C, D).

In this work, emphasis was also centered on the associations between the biodiversity of each module and single ecosystem functions. The biodiversity within Modules 2–4 was positively correlated with the activity of phosphatase, urease, and β-glucosidase, whereas it was negatively correlated with the activity of saccharase (*P* < 0.01) (Figure 4E). Besides, the accumulation of TP and NH_4_^+^ showed significantly negative correlations (*P* < 0.01) with the biodiversity of Modules 2–4. The accumulation of TN, NO_3_^–^ and Fe-element cycling was positively related to the biodiversity of Module 2 but not significantly associated with that of most other modules (Figure 4E). No correlation was observed between the accumulation of SOC, DOC, and AS and the diversity of Modules 1–4 (Figure 4E). Interestingly, the significant relationship between the effects of Module 1 on single ecosystem functions appeared to be the exact reverse of Modules 2–4 (Figure 4E).

**FIGURE 5:**
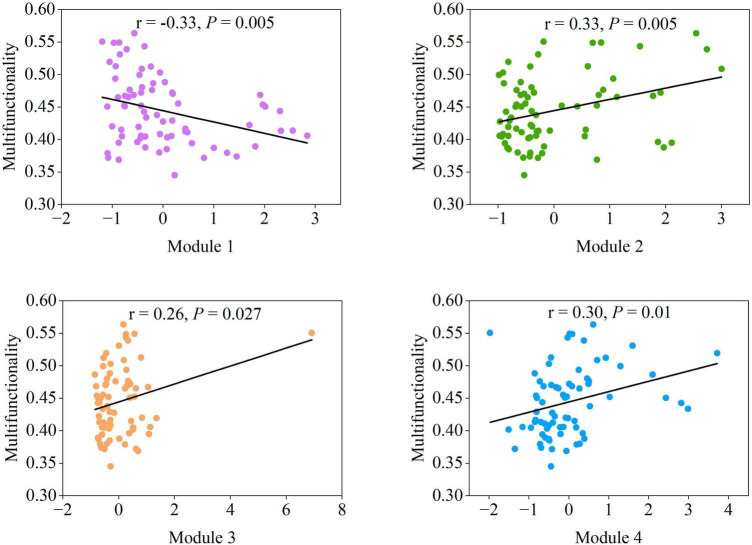
The correlation between the four main modules and multifunctionality. Statistical analysis was performed using ordinary least squares linear regressions.

The relationships between each module and multifunctionality were evaluated to further investigate the mechanism of the modules on adjusting soil multifunctionality. Significant positive correlations (*P* < 0.05) between the diversity of Modules 2–4 and soil multifunctionality were detected (Figure 5). The richness of Module 1 was the only exception, presenting a significantly negative relationship (*P* < 0.01) (Figure 5). The specificity of Module 1 (Figure 5) and the characteristics of the microbial co-occurrence networks (Figures 3, 4) indicated that soil microbial taxa in modules regulate multifunctionality. We next explored the importance of dominant taxa within ecological modules as manipulators of soil ecosystem multifunctionality.

**FIGURE 6:**
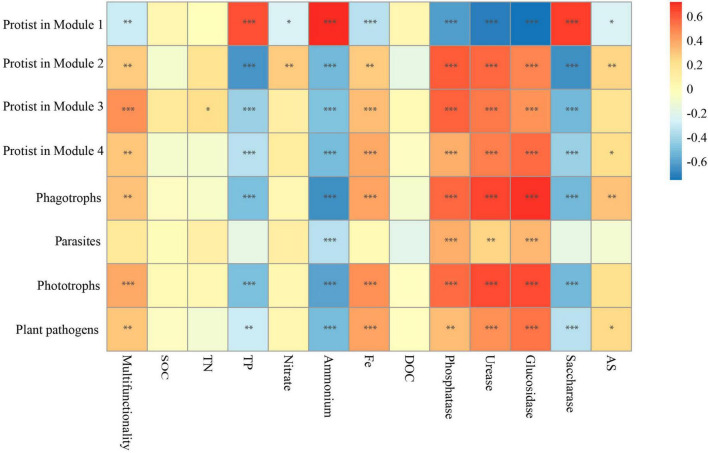
The correlation between the main protists in four modules, protistan functional groups, and multifunctionality. Significant correlations (Spearman, ρ < 0.05) between protists within each module, as well as between different protistan functional groups and ecosystem functions. *P-*values were indicated by asterisks: *P < 0.05, **P < 0.01, ***P < 0.001, and ****P < 0.0001.

Given that the effects of protists on the majority of single functions were consistent with most modules (Figures 2E, 4E), and protists showed specificity in their impacts on soil multifunctionality (Figure 2C and Supplementary Figure 3), we speculated that protists in the modules were the dominant taxa regulating soil multifunctionality. The associations between protistan diversity within each module and soil multifunctionality were then tested. The richness of protists within Modules 2, 3, and 4 was positively related to the multifunctionality (Figure 6). By contrast, the protistan diversity in Module 1 had a negative association with the multifunctionality (Figure 6). The impacts of protists within modules on soil multifunctionality were coherent with those of each module (Figures 5, 6). Besides, we assigned the protists into various functional groups based on their nutrient-uptake mode (Xiong et al., 2020), including phagotrophs, parasites, phototrophs, and plant pathogens, and tested their associations with single functions. Protists, especially microbial-feeding phagotrophs, are significantly correlated with soil multifunctionality (Figure 6). The phagotrophic protists exerted a significant impact on both enzyme activity and the supply of nitrogen and phosphorus nutrients (Figure 6). These results provided independent evidence for the hypothesis of protists regulating multifunctionality.

### 3.4 Accounting for multiple multifunctionality drivers

**FIGURE 7:**
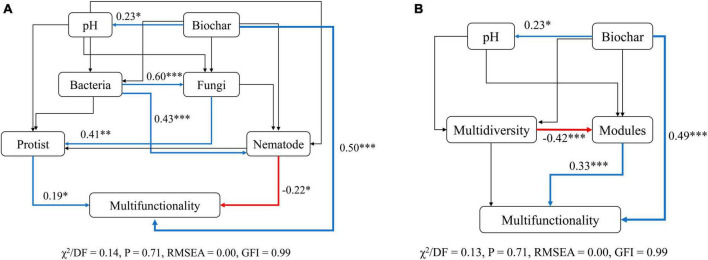
Structural equation models (SEMs) accounting for the direct and indirect relationships between biochar, modules, soil biodiversity, and multifunctionality. **(A)** Structural equation model describing the relationship between soil biodiversity (bacteria, fungi, protists, and nematode) and multifunctionality. **(B)** Structural equation model describing the correlation between modules and multifunctionality. The blue and red lines indicate positive and opposite effects, respectively. The black lines show no significant impact. The thickness of the line infers the strength of the relationship. Asterisks indicate the significance level of each coefficient: *P*-values: *p < 0.05, **p < 0.01, and ***p < 0.001.

Structural equation models (SEMs) were then conducted to infer the multiple direct and indirect impacts of biochar amendment, microbial diversity, and modules in explaining soil ecosystem multifunctionality. To test whether various indirect pathways drive the biochar-biodiversity-multifunctionality relationships, we divided the microbial diversity into multidiversity, modules and different soil microbes, including bacteria, fungi, protists, and nematodes, and performed SEMs for both cases separately. Our results from both cases suggested that the direct positive impacts (*P* < 0.001) of biochar amendment on soil ecosystem multifunctionality were maintained even after accounting for the effects of other ecosystem factors simultaneously (Figure 7). The biochar amendment was the predominant driver of soil multifunctionality and positively related to soil pH which had limited contribution to multifunctionality indirectly (Figure 7). Consistent with the results shown in Figure 2A and Supplementary Table 1, SEMs for soil taxonomic microorganisms revealed that the diversity of nematodes was negatively correlated with soil multifunctionality, whereas that of protists was positively correlated with soil multifunctionality (*P* < 0.05) (Figure 7A). More importantly, although the bacterial and fungal diversity did not affect soil multifunctionality directly, they did affect the multifunctionality indirectly by altering the diversity of nematodes and protists, respectively (Figure 7A). In addition, modules had direct and significant positive effects (*P* < 0.001) on soil multifunctionality (Figure 7B). Multidiversity, however, indirectly influenced soil ecosystem multifunctionality primarily by negatively affecting the modules (*R* = −0.42, *P* < 0.001) (Figure 7B).

In summary, these results demonstrated that the effects of multidiversity on multifunctionality were indirectly driven by changes in modules. The association of the diversity of soil higher trophic level microorganisms with soil ecosystem multifunctionality was stronger and more significant than that of the lower ones.

## 4 Discussion

### 4.1 Effects of biochar on multifunctionality in paddy soil

Previous studies have confirmed that biochar addition actively enhanced the soil ecosystem functions (Liang et al., 2017; Dong et al., 2022; Han et al., 2022; Ren et al., 2023). In this study, we also found that the different gradients of biochar amendment improved the soil multifunctionality in the early stage (*P* < 0.01) (Figure 1A). This was largely attributed to the biochar addition increasing soil pH, thus hindering soil acidification (Dong et al., 2022; Figure 7). Soil acidification decelerated nutrient provisioning, litter decomposition, and element cycling, thereby negatively affecting soil ecosystem multifunctionality (Eldridge et al., 2020; Shen et al., 2021; Wei et al., 2022). In addition, with its highly porous and loose characteristics, biochar can promote the growth and proliferation of soil microorganisms by offering more room (Jaafar et al., 2014, 2015; Ye et al., 2017). Furthermore, biochar may enhance soil multifunctionality by improving soil biological properties, such as boosting enzyme activities. Soil enzyme activities are crucial indicators that should be taken into consideration in evaluating the soil ecosystem multifunctionality. Wang et al. (2015) reported that the activities of some extracellular enzymes involved in nutrient cycling were increased in biochar-amended fluvo-aquic soil (Wang et al., 2015). However, the impact of biochar addition on soil multifunctionality was weakened in the late stage, which mainly accounted for the buffering capacity of paddy soils. The soil buffering capacity alleviated the pH enhancement induced by the biochar amendments (Li et al., 2019). Simultaneously, Xu et al. (2012) revealed that the biochar application led to an improvement in the soil buffering capacity (Xu et al., 2012). These results reflected that the biochar amendment on paddy soil exhibited the buffering capacity, which in turn increased with adding biochar. The buffering capacity, nevertheless, was not immediately noticeable in the early stage, while only became evident in the later stage. This supports our first hypothesis that biochar positively affects soil multifunctionality by increasing soil pH, but this effect faded owing to the soil buffering capacity at the late stage. Moreover, aggregate size was closely related to biodiversity levels in biochar-amended paddy soils, with smaller aggregates supporting a higher diversity of soil organisms (Supplementary Figure 2). This indicated that smaller soil aggregates exhibited higher biodiversity compared to larger aggregates. The microscale spatial heterogeneity and complexity of soil aggregates impacted the distribution of microorganisms, concurrently influencing microbial diversity in biochar-amended paddy soils (Han et al., 2021).

### 4.2 Microbial diversity and ecosystem multifunctionality

The biodiversity of soil protists was positively related to soil multifunctionality, whereas that of bacteria, fungi, and nematodes exhibited negative correlations with multifunctionality (Figures 2A–D and Supplementary Figure 3). SEMs further revealed that the direct associations between the diversity of protists and nematodes and soil multifunctionality were robust after accounting for four soil microorganisms simultaneously. By contrast, bacterial and fungal diversity was indirectly correlated with multifunctionality (Figure 7B). This is corresponding to our second hypothesis that the relationship between the biodiversity of soil higher trophic level microorganisms and soil ecosystem multifunctionality was stronger and more significant than that of the lower ones. Moreover, multiple potential associations between soil microbial biodiversity impacted ecosystem multifunctionality (Delgado-Baquerizo et al., 2020). Specifically, the diversity of protists and nematodes showed a positive association with that of fungi and bacteria, respectively (*P* < 0.01) (Figure 7B), indicating the presence of potential predator-prey relationships. Bacteria and fungi may indirectly impact multifunctionality by being preyed upon by protists and nematodes (Han et al., 2022). For instance, the negative relationships (*P* < 0.01) between the typical nematodes (Aracolaimida and Chromadorida) and multifunctionality were observed (Supplementary Table 1). According to the SEM, there was a positive correlation between the diversity of nematodes and that of bacteria (Figure 7B). Considering these associations and interactions among different trophic levels, it could indicate that nematodes preying on bacteria involved in soil functions lead to a significant negative correlation between nematodes and multifunctionality. This is aligned with the earlier finding of Wagg et al. (2019), revealing that the interactions among the multitrophic members of soil microbiome also have contributed to soil ecosystem functions directly or indirectly (Wagg et al., 2019).

Overall, the alterations in soil multifunctionality exhibited a negative association with the average of soil biodiversity (multidiversity) (Supplementary Figure 4), indicating that changes in soil microbial diversity negatively impact soil ecosystem multifunctionality. Currently, the effects of soil microbial diversity on ecosystem processes remain largely controversial because the positive, negative, and neutral relationships between soil multidiversity and multifunctionality have been reported (Nielsen et al., 2011; Bradford et al., 2014; Wagg et al., 2014; Delgado-Baquerizo et al., 2016, 2020; Chen et al., 2020; Dong et al., 2022; Jiao et al., 2022; Fan et al., 2023). These contradictory findings reported in prior studies may be attributed to two main reasons: (1) environmental change factors, including biotic and abiotic factors, can alter the effects of soil diversity on soil ecosystem functions (Hu et al., 2021; Yang et al., 2022); and (2) the soil biodiversity-multifunctionality relationships may be contingent upon the number of functions and their combinations (Bradford et al., 2014; Lefcheck et al., 2015; Soliveres et al., 2016; Chen et al., 2020). All soil ecosystem functions have the potential to be either positively or negatively influenced by soil diversity or the interplay among the functions (Kareiva et al., 2007; Lefcheck et al., 2015). As more functions and trophic levels are considered, the biodiversity-multifunctionality relationships become more comprehensive and persuasive (Soliveres et al., 2016; Wang et al., 2019). Thus, the soil biodiversity-multifunctionality relationships shift as the spatial-temporal environmental conditions change. Different ecosystems have distinct purposes and provide various services; hence, the results of measuring the relationship above vary in different ecosystems (Jiao et al., 2022; Fan et al., 2023).

### 4.3 Microbial modules and interactions impact ecosystem functions

The correlations of module diversity with soil single functions were stronger and more significant than those of soil microbial taxa (Figures 2E, 4E). Enzyme activities and the accumulation of NH_4_^+^ and TP were significantly associated with the diversity of modules (Figure 4E). The stronger relationships between modules and single functions are probably explained by the fact that the ecological clusters can support similar functions (Jiao et al., 2022). Theoretically, species with similar ecological preferences are more likely to cluster and form modules that promote similar functions (Delgado-Baquerizo et al., 2020; Wang et al., 2022). Interactions among soil horizontal and vertical species lead to altering ecosystem functions (Duffy et al., 2007). The microbial co-occurrence networks were dominated by positive correlations (Figure 3A), suggesting potential cooperative interactions among taxa within modules. The cooperative interactions among the species in modules result in their synergistic and complementary relationships that consistently enhance the impact of modules on various ecosystem functions (Chen et al., 2022). No correlation, however, was found between some single ecosystem functions (including the accumulation of TN, SOC, AS, and DOC) and the diversity of modules (Figure 4E). This discrepancy is largely triggered by functional redundancy as soil microbial communities may have overlapping functions (Allison and Martiny, 2008), implying that multiple taxonomic groups can perform the same ecosystem functions (Li et al., 2021). However, the existence of functional redundancy in ecosystems affects evaluating the relationships between biodiversity and soil ecosystem functions. Microbial communities display highly functional redundancy for some basic functions, such as microbial respiration, whereas redundancy may not be observed for specific or specialized functions (Langenheder et al., 2006; Reich et al., 2012; Chen et al., 2020). The functional redundancy tends to fade as more functions are measured (Hector and Bagchi, 2007; Isbell et al., 2011). It also explains why multitrophic levels and more appropriate functions are needed to be considered in exploring the mechanism of biodiversity-multifunctionality relationships.

Four primary modules were detected based on the soil co-occurrence network of soil organisms to reveal how soil modules regulate the soil ecosystem multifunctionality. Soil microbial community diversity and multifunctionality exhibited a positive correlation in Module 2–4 (*P* < 0.05), yet a negative correlation in Module 1 (*P* < 0.01) (Figure 5). The specificity of Module 1 has been primarily attributed to closer and more complicated connectedness and more robust interactions between species of Module 1 than other modules (Figure 3A). This result revealed that trade-offs exist when measuring soil biodiversity-multifunctionality relationships (Byrnes et al., 2014; Hu et al., 2021). The negative relationships and complicated interactions within Module 1 reflected an increasing frequency of trade-offs among ecosystem functions as more species and their interactions were considered (Lefcheck et al., 2015).

According to Fan et al. (2021), keystone taxa within modules played a pivotal role in regulating the ecosystem functions of terrestrial ecosystems subjected to 40 years of fertilization (Fan et al., 2021). Keystone taxa, integral to the flow of energy and materials, substantially contribute to the productivity of the soil ecosystem in ecological networks (Toju et al., 2018; Shi et al., 2020). Protists in Module 1 exhibited a negative correlation with multifunctionality, suggesting a unique interaction dynamic within this module that may involve complex trade-offs. Conversely, protists in Modules 2, 3, and 4 showed positive correlations with multifunctionality, highlighting their fundamental contribution to promoting a range of soil functions (Figure 6). Therefore, we identified the protistan keystone taxa in the modules based on the microbial co-occurrence network analysis (Figures 4A–D). Moreover, the major phyla of predatory protists, such as Amoebozoa, Stramenopiles, Excavata, and Alveolata, were found to positively correlate with ecosystem multifunctionality (Supplementary Table 1). The predation or grazing of protists was essential for directing carbon and energy flow toward higher trophic levels while facilitating the release of dissolved nutrients throughout the food webs (Bjorbaekmo et al., 2020). Guo et al. (2022) demonstrated that predatory protists interacted with bacteria in the plant-soil microbiome, ultimately augmenting plant health and crop yields (Guo et al., 2022). In our study, the biochar amendment of paddy soil enhanced the abundance of predatory protists (Asiloglu et al., 2021) and played a critical role in maintaining ecosystem functioning.

The SEMs demonstrated that the modules exerted a significant effect on soil multifunctionality (*P* < 0.001), while multidiversity indirectly affected multifunctionality through its impact on modules (*P* < 0.001) (Figure 7A). Simultaneously, the influence of modules on soil multifunctionality surpassed that of individual microorganisms (Figure 7B). Recent research also revealed that modules regulated soil multifunctionality more remarkably than individual microorganisms and multidiversity (Delgado-Baquerizo et al., 2020; Jiao et al., 2022; Yang et al., 2022). These findings convincingly illustrated the crucial role of modules formed by soil microbes with similar ecological preferences as critical drivers of ecosystem functioning. Together, our study highlights the more prominent role of modules in determining soil multifunctionality.

## 5 Conclusion

Collectively, our work supports the claim that biochar amendment promotes the ecosystem multifunctionality of paddy soil, while the effects were diminished in the late stage. The soil taxonomic diversity and core modules are tied to the ecosystem functioning in biochar-amended paddy soil, including multifunctionality and multiple individual functions. The core taxa within modules in biochar-amended paddy soil serves as the best predictor for ecosystem functioning. These modules’ interactions and interrelations are crucial in maintaining and enhancing soil multifunctionality, highlighting the significance of microbial community structure in ecosystem function. This observation underlines the critical role of module-based biodiversity in determining the ecological outcomes and reinforces the importance of understanding the complex interactions among different functional groups within these modules for effective ecosystem management.

## Data availability statement

The datasets presented in this study can be found in online repositories. The names of the repository/repositories and accession number(s) can be found in the article/Supplementary material.

## Author contributions

YX: Writing – original draft, Writing – review and editing. GZ: Data curation, Visualization, Writing – review and editing. XQ: Supervision, Writing – review and editing. FL: Writing – review and editing. LC: Writing – review and editing. JZ: Writing – review and editing, Funding acquisition, Supervision.

## References

[B1] Allison S. D. Martiny J. B. (2008). Resistance, resilience, and redundancy in microbial communities. *Proc. Natl. Acad. Sci. U.S.A.* 105 11512–11519. 10.1073/pnas.0801925105 18695234 PMC2556421

[B2] Asiloglu R. Shiroishi K. Suzuki K. Turgay O. C. Harada N. (2021). Soil properties have more significant effects on the community composition of protists than the rhizosphere effect of rice plants in alkaline paddy field soils. *Soil Biol. Biochem.* 161:108397. 10.1016/j.soilbio.2021.108397

[B3] Bardgett R. D. Van Der Putten W. H. (2014). Belowground biodiversity and ecosystem functioning. *Nature* 515 505–511. 10.1038/nature13855 25428498

[B4] Bjorbaekmo M. F. Evenstad A. Rosaeg L. L. Krabberod A. K. Logares R. (2020). The planktonic protist interactome: Where do we stand after a century of research? *ISME J.* 14 544–559. 10.1038/s41396-019-0542-5 31685936 PMC6976576

[B5] Bradford M. A. Wood S. A. Bardgett R. D. Black H. I. Bonkowski M. Eggers T. (2014). Discontinuity in the responses of ecosystem processes and multifunctionality to altered soil community composition. *Proc. Natl. Acad. Sci. U.S.A.* 111 14478–14483. 10.1073/pnas.1413707111 25246582 PMC4210050

[B6] Byrnes J. E. Gamfeldt L. Isbell F. Lefcheck J. S. Griffin J. N. Hector A. (2014). Investigating the relationship between biodiversity and ecosystem multifunctionality: Challenges and solutions. *Methods Ecol. Evol.* 5 111–124. 10.1111/2041-210x.12143

[B7] Chen Q. Ding J. Zhu D. Hu H. Delgado-Baquerizo M. Ma Y. (2020). Rare microbial taxa as the major drivers of ecosystem multifunctionality in long-term fertilized soils. *Soil Biol. Biochem.* 141:107686. 10.1016/j.soilbio.2019.107686

[B8] Chen W. Wang J. Chen X. Meng Z. Xu R. Duoji D. (2022). Soil microbial network complexity predicts ecosystem function along elevation gradients on the Tibetan Plateau. *Soil Biol. Biochem.* 172:108766. 10.1016/j.soilbio.2022.108766

[B9] Coban O. De Deyn G. B. Van Der Ploeg M. (2022). Soil microbiota as game-changers in restoration of degraded lands. *Science* 375:abe0725. 10.1126/science.abe0725 35239372

[B10] Delgado-Baquerizo M. Maestre F. T. Reich P. B. Jeffries T. C. Gaitan J. J. Encinar D. (2016). Microbial diversity drives multifunctionality in terrestrial ecosystems. *Nat. Commun.* 7:10541. 10.1038/ncomms10541 26817514 PMC4738359

[B11] Delgado-Baquerizo M. Reich P. B. Trivedi C. Eldridge D. J. Abades S. Alfaro F. D. (2020). Multiple elements of soil biodiversity drive ecosystem functions across biomes. *Nat. Ecol. Evol.* 4 210–220. 10.1038/s41559-019-1084-y 32015427

[B12] Dong Z. Li H. Xiao J. Sun J. Liu R. Zhang A. (2022). Soil multifunctionality of paddy field is explained by soil pH rather than microbial diversity after 8-years of repeated applications of biochar and nitrogen fertilizer. *Sci. Total Environ.* 853:158620. 10.1016/j.scitotenv.2022.158620 36084779

[B13] Duffy J. E. Cardinale B. J. France K. E. Mcintyre P. B. Thebault E. Loreau M. (2007). The functional role of biodiversity in ecosystems: Incorporating trophic complexity. *Ecol. Lett.* 10 522–538. 10.1111/j.1461-0248.2007.01037.x 17498151

[B14] Eldridge D. J. Delgado-Baquerizo M. Quero J. L. Ochoa V. Gozalo B. García-Palacios P. (2020). Surface indicators are correlated with soil multifunctionality in global drylands. *J. Appl. Ecol.* 57 424–435. 10.1111/1365-2664.13540

[B15] Elliott E. T. (1986). Aggregate structure and carbon, nitrogen, and phosphorus in native and cultivated soils. *Soil Sci. Soc. Am. J.* 50 627–633. 10.2136/sssaj1986.03615995005000030017x

[B16] Fan K. Chu H. Eldridge D. J. Gaitan J. J. Liu Y. R. Sokoya B. (2023). Soil biodiversity supports the delivery of multiple ecosystem functions in urban greenspaces. *Nat. Ecol. Evol.* 7 113–126. 10.1038/s41559-022-01935-4 36631668

[B17] Fan K. Delgado-Baquerizo M. Guo X. Wang D. Zhu Y. G. Chu H. (2021). Biodiversity of key-stone phylotypes determines crop production in a 4-decade fertilization experiment. *ISME J.* 15 550–561. 10.1038/s41396-020-00796-8 33028975 PMC8027226

[B18] Fierer N. (2017). Embracing the unknown: Disentangling the complexities of the soil microbiome. *Nat. Rev. Microbiol.* 15 579–590. 10.1038/nrmicro.2017.87 28824177

[B19] Guerra C. A. Bardgett R. D. Caon L. Crowther T. W. Delgado-Baquerizo M. Montanarella L. (2021). Tracking, targeting, and conserving soil biodiversity. *Science* 371 239–241. 10.1126/science.abd7926 33446546

[B20] Guo S. Tao C. Jousset A. Xiong W. Wang Z. Shen Z. (2022). Trophic interactions between predatory protists and pathogen-suppressive bacteria impact plant health. *ISME J.* 16 1932–1943. 10.1038/s41396-022-01244-5 35461357 PMC9296445

[B21] Han S. Delgado-Baquerizo M. Luo X. Liu Y. Van Nostrand J. D. Chen W. (2021). Soil aggregate size-dependent relationships between microbial functional diversity and multifunctionality. *Soil Biol. Biochem.* 154:108143. 10.1016/j.soilbio.2021.108143

[B22] Han Z. Xu P. Li Z. Lin H. Zhu C. Wang J. (2022). Microbial diversity and the abundance of keystone species drive the response of soil multifunctionality to organic substitution and biochar amendment in a tea plantation. *GCB Bioenergy* 14 481–495. 10.1111/gcbb.12926

[B23] Hector A. Bagchi R. (2007). Biodiversity and ecosystem multifunctionality. *Nature* 448 188–U186. 10.1038/nature05947 17625564

[B24] Hu W. Ran J. Dong L. Du Q. Ji M. Yao S. (2021). Aridity-driven shift in biodiversity-soil multifunctionality relationships. *Nat. Commun.* 12:5350. 10.1038/s41467-021-25641-0 34504089 PMC8429721

[B25] Isbell F. Calcagno V. Hector A. Connolly J. Harpole W. S. Reich P. B. (2011). High plant diversity is needed to maintain ecosystem services. *Nature* 477 199–U196. 10.1038/nature10282 21832994

[B26] Jaafar N. M. Clode P. L. Abbott L. K. (2014). Microscopy observations of habitable space in biochar for colonization by fungal hyphae from soil. *J. Integr. Agric.* 13 483–490. 10.1016/s2095-3119(13)60703-0

[B27] Jaafar N. M. Clode P. L. Abbott L. K. (2015). Soil microbial responses to biochars varying in particle size surface and pore properties. *Pedosphere* 25 770–780. 10.1016/s1002-0160(15)30058-8

[B28] Jansson J. K. Hofmockel K. S. (2020). Soil microbiomes and climate change. *Nat. Rev. Microbiol.* 18 35–46. 10.1038/s41579-019-0265-7 31586158

[B29] Jiao S. Lu Y. Wei G. (2022). Soil multitrophic network complexity enhances the link between biodiversity and multifunctionality in agricultural systems. *Glob. Chang. Biol.* 28 140–153. 10.1111/gcb.15917 34610173

[B30] Kareiva P. Watts S. Mcdonald R. Boucher T. (2007). Domesticated nature: Shaping landscapes and ecosystems for human welfare. *Science* 316 1866–1869. 10.1126/science.1140170 17600209

[B31] Langenheder S. Lindstrom E. S. Tranvik L. J. (2006). Structure and function of bacterial communities emerging from different sources under identical conditions. *Appl. Environ. Microbiol.* 72 212–220. 10.1128/aem.72.1.212-220.2006 16391045 PMC1352196

[B32] Lefcheck J. S. Byrnes J. E. Isbell F. Gamfeldt L. Griffin J. N. Eisenhauer N. (2015). Biodiversity enhances ecosystem multifunctionality across trophic levels and habitats. *Nat. Commun.* 6:6936. 10.1038/ncomms7936 25907115 PMC4423209

[B33] Lehmann J. Bossio D. A. Kogel-Knabner I. Rillig M. C. (2020). The concept and future prospects of soil health. *Nat. Rev. Earth Environ.* 1 544–553. 10.1038/s43017-020-0080-8 33015639 PMC7116140

[B34] Li Y. C. Li Y. F. Chang S. X. Yang Y. F. Fu S. L. Jiang P. K. (2018). Biochar reduces soil heterotrophic respiration in a subtropical plantation through increasing soil organic carbon recalcitrancy and decreasing carbon degrading microbial activity. *Soil Biol. Biochem.* 122 173–185. 10.1016/j.soilbio.2018.04.019

[B35] Li Y. Ge Y. Wang J. Shen C. Wang J. Liu Y. (2021). Functional redundancy and specific taxa modulate the contribution of prokaryotic diversity and composition to multifunctionality. *Mol. Ecol.* 30 2915–2930. 10.1111/mec.15935 33905157

[B36] Li Z. Unzué-Belmonte D. Cornelis J. Linden C. V. Struyf E. Ronsse F. (2019). Effects of phytolithic rice-straw biochar, soil buffering capacity and pH on silicon bioavailability. *Plant Soil* 438 187–203. 10.1007/s11104-019-04013-0

[B37] Liang Y. Pei M. Wang D. Cao S. Xiao X. Sun B. (2017). Improvement of soil ecosystem multifunctionality by dissipating manure-induced antibiotics and resistance genes. *Environ. Sci. Technol.* 51 4988–4998. 10.1021/acs.est.7b00693 28394116

[B38] Liu Y. Lonappan L. Brar S. K. Yang S. (2018). Impact of biochar amendment in agricultural soils on the sorption, desorption, and degradation of pesticides: A review. *Environ. Sci. Technol.* 645 60–70. 10.1016/j.scitotenv.2018.07.099 30015119

[B39] Nielsen U. N. Ayres E. Wall D. H. Bardgett R. D. (2011). Soil biodiversity and carbon cycling: A review and synthesis of studies examining diversity-function relationships. *Eur. J. Soil Sci.* 62 105–116. 10.1111/j.1365-2389.2010.01314.x

[B40] Palansooriya K. N. Wong J. T. Hashimoto Y. Huang L. Rinklebe J. Chang S. X. (2019). Response of microbial communities to biochar-amended soils: A critical review. *Biochar* 1 3–22. 10.1007/s42773-019-00009-2

[B41] Reich P. B. Tilman D. Isbell F. Mueller K. Hobbie S. E. Flynn D. F. (2012). Impacts of biodiversity loss escalate through time as redundancy fades. *Science* 336 589–592. 10.1126/science.1217909 22556253

[B42] Ren T. Liao J. Jin L. Delgado-Baquerizo M. Ruan H. (2023). Application of biogas-slurry and biochar improves soil multifunctionality in a poplar plantation during afforestation processes. *J. Plant Soil* 1–17. 10.1007/s11104-023-05968-x

[B43] Shen Y. Tian D. Hou J. Wang J. Zhang R. Li Z. (2021). Forest soil acidification consistently reduces litter decomposition irrespective of nutrient availability and litter type. *Funct. Ecol.* 35 2753–2762. 10.1111/1365-2435.13925

[B44] Shi Y. Delgado-Baquerizo M. Li Y. Yang Y. Zhu Y. G. Penuelas J. (2020). Abundance of kinless hubs within soil microbial networks are associated with high functional potential in agricultural ecosystems. *Environ. Int.* 142:105869. 10.1016/j.envint.2020.105869 32593837

[B45] Soliveres S. Van Der Plas F. Manning P. Prati D. Gossner M. M. Renner S. C. (2016). Biodiversity at multiple trophic levels is needed for ecosystem multifunctionality. *Nature* 536 456–459. 10.1038/nature19092 27533038

[B46] Toju H. Peay K. G. Yamamichi M. Narisawa K. Hiruma K. Naito K. (2018). Core microbiomes for sustainable agroecosystems. *Nat. Plants* 4 247–257. 10.1038/s41477-018-0139-4 29725101

[B47] Wagg C. Bender S. F. Widmer F. Van Der Heijden M. G. (2014). Soil biodiversity and soil community composition determine ecosystem multifunctionality. *Proc. Natl. Acad. Sci. U.S.A.* 111 5266–5270. 10.1073/pnas.1320054111 24639507 PMC3986181

[B48] Wagg C. Schlaeppi K. Banerjee S. Kuramae E. E. Van Der Heijden M. G. (2019). Fungal-bacterial diversity and microbiome complexity predict ecosystem functioning. *Nat. Commun.* 10:4841. 10.1038/s41467-019-12798-y 31649246 PMC6813331

[B49] Wang L. Delgado-Baquerizo M. Wang D. Isbell F. Liu J. Feng C. (2019). Diversifying livestock promotes multidiversity and multifunctionality in managed grasslands. *Proc. Natl. Acad. Sci. U.S.A.* 116 6187–6192. 10.1073/pnas.1807354116 30850539 PMC6442565

[B50] Wang X. Song D. Liang G. Zhang Q. Ai C. Zhou W. (2015). Maize biochar addition rate influences soil enzyme activity and microbial community composition in a fluvo-aquic soil. *Appl. Soil Ecol.* 96 265–272. 10.1016/j.apsoil.2015.08.018

[B51] Wang Y. F. Chen P. Wang F. H. Han W. X. Qiao M. Dong W. X. (2022). The ecological clusters of soil organisms drive the ecosystem multifunctionality under long-term fertilization. *Environ. Int.* 161:107133. 10.1016/j.envint.2022.107133 35149447

[B52] Wei Y. Jing X. Su F. Li Z. Wang F. Guo H. (2022). Does pH matter for ecosystem multifunctionality? An empirical test in a semi-arid grassland on the Loess Plateau. *Funct. Ecol.* 36 1739–1753. 10.1111/1365-2435.14057

[B53] Xiong W. Song Y. Yang K. Gu Y. Wei Z. Kowalchuk G. A. (2020). Rhizosphere protists are key determinants of plant health. *Microbiome* 8:27. 10.1186/s40168-020-00799-9 32127034 PMC7055055

[B54] Xu R. Zhao A. Yuan J. Jiang J. (2012). pH buffering capacity of acid soils from tropical and subtropical regions of China as influenced by incorporation of crop straw biochars. *J. Soils Sediments* 12 494–502. 10.1007/s11368-012-0483-3

[B55] Yang G. Ryo M. Roy J. Lammel D. R. Ballhausen M. B. Jing X. (2022). Multiple anthropogenic pressures eliminate the effects of soil microbial diversity on ecosystem functions in experimental microcosms. *Nat. Commun.* 13:4260. 10.1038/s41467-022-31936-7 35871070 PMC9308766

[B56] Yao Y. Shao M. Fu X. Wang X. Wei X. (2019). Effects of shrubs on soil nutrients and enzymatic activities over a 0–100 cm soil profile in the desert-loess transition zone. *Catena* 174 362–370. 10.1016/j.catena.2018.11.031

[B57] Ye J. Joseph S. D. Ji M. Nielsen S. Mitchell D. R. Donne S. (2017). Chemolithotrophic processes in the bacterial communities on the surface of mineral-enriched biochars. *ISME J.* 11 1087–1101. 10.1038/ismej.2016.187 28169988 PMC5437921

[B58] Zheng H. Wang X. Luo X. Wang Z. Xing B. (2018). Biochar-induced negative carbon mineralization priming effects in a coastal wetland soil: Roles of soil aggregation and microbial modulation. *Sci. Total Environ.* 610-611 951–960. 10.1016/j.scitotenv.2017.08.166 28830055

[B59] Zhou G. Xu X. Qiu X. Zhang J. (2019). Biochar influences the succession of microbial communities and the metabolic functions during rice straw composting with pig manure. *Bioresour. Technol.* 272 10–18. 10.1016/j.biortech.2018.09.135 30292912

