# Supplementary material for: Biodiversity of network modules drives ecosystem functioning in biochar-amended paddy soil
Source: Front Microbiol. 2024 Jan 24;15:1341251. doi: 10.3389/fmicb.2024.1341251 (PMC10847562; doi:10.3389/fmicb.2024.1341251)
Supplement: Supplementary file 1 [file Data_Sheet_1.docx]

Supplementary Material

**Protists as the major drivers of ecosystem functioning in** **biochar-amended paddy soil**

**Yu Xiao^1,2,4^, Guixiang Zhou^1,4^, Xiuwen Qiu^3^, Fangming Liu****^1,2^, Lin Chen^1^, Jiabao Zhang^1*^**

^1^ State Key Laboratory of Soil and Sustainable Agriculture, Institute of Soil Science, Chinese Academy of Sciences, Nanjing, 210008, China

^2^ University of Chinese Academy of Sciences, Beijing, China

^3^ College of Landscape Architecture, Jiangsu Vocational College of Agriculture and Forestry, Jurong, 212400, China

^4^ These authors contributed equally: Yu Xiao, Guixiang Zhou

***Correspondence:**

Prof. Jiabao Zhang.

E-mail: jbzhang@issas.ac.cn

Tel: +86-25-8688-1228

Fax: +86-25-8688-1000

Table S1. The correlation between the main protists (Alveolata, Amoebozoa, Excavata and Stramenopiles), two nematodes (Araeolaimida and Chromadorida) and multifunctionality.

| The main protists | | | The main nematodes | | |
| --- | --- | --- | --- | --- | --- |
|  | r | *P* |  | r | *P* |
| Alveolata | 0.26 | 0.026* | Araeolaimida | -0.35 | 0.003** |
| Amoebozoa | 0.40 | <0.001*** | Chromadorida | -0.35 | 0.003** |
| Excavata | 0.29 | 0.013* |  |  |  |
| Stramenopiles | 0.27 | 0.023* |  |  |  |


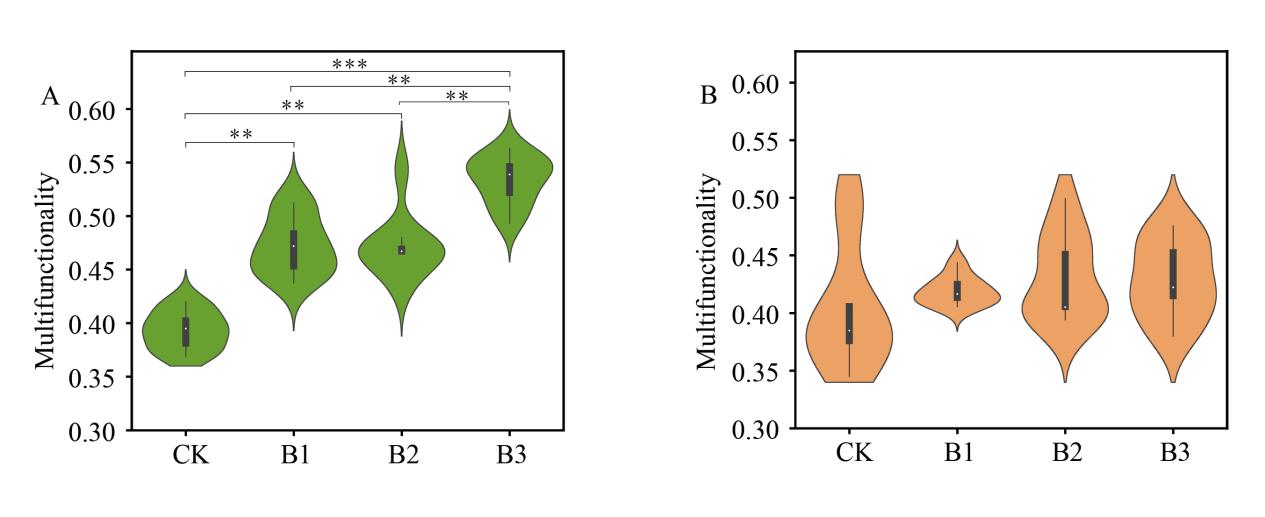


Fig. S1 The multifunctionality in different levels of biochar addition. A: early stage, B: late stage. Abbreviations: CK, control, and without additive; B1, with the addition of 2.5 g kg^-1^ biochar; B2, with the addition of 5 g kg^-1^ biochar; B3, with the addition of 10 g kg^-1^ biochar.


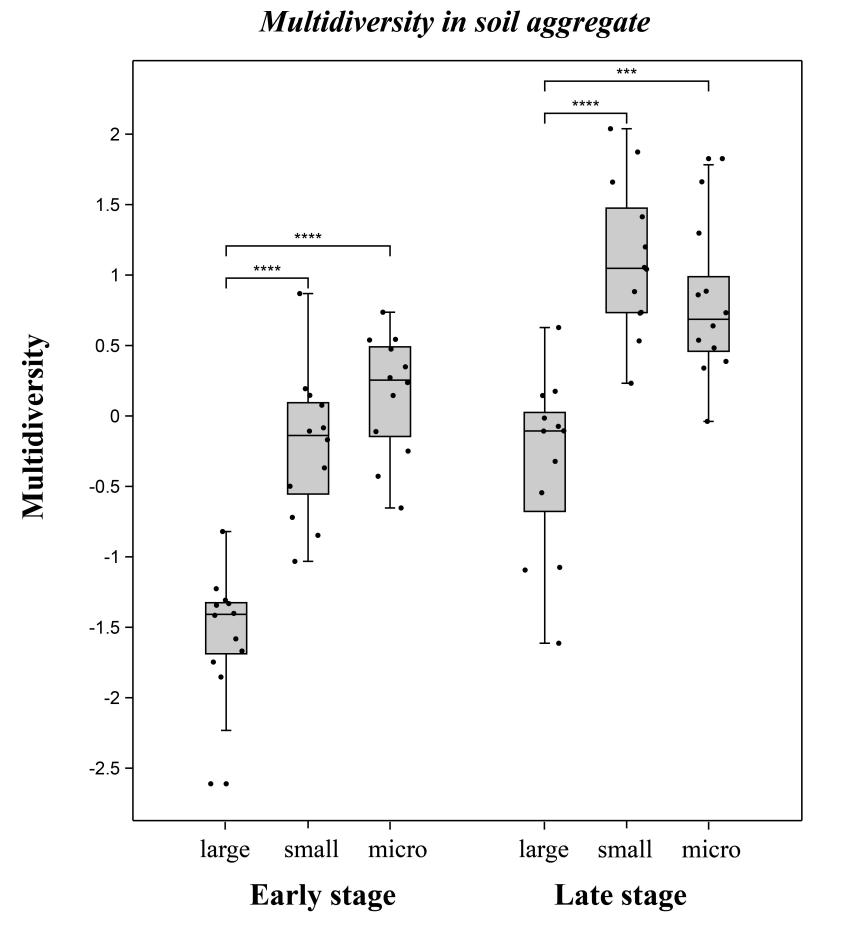


Fig. S2 The multidiversity (the biodiversity of selected groups of soil organisms (averaged standardized between 0 and 1)) at different temporal aggregation levels. Statistical analysis was performed using ordinary least squares linear regressions; P values were indicated by asterisks: *P < 0.05, **P < 0.01, ***P < 0.001, and ****P < 0.0001.


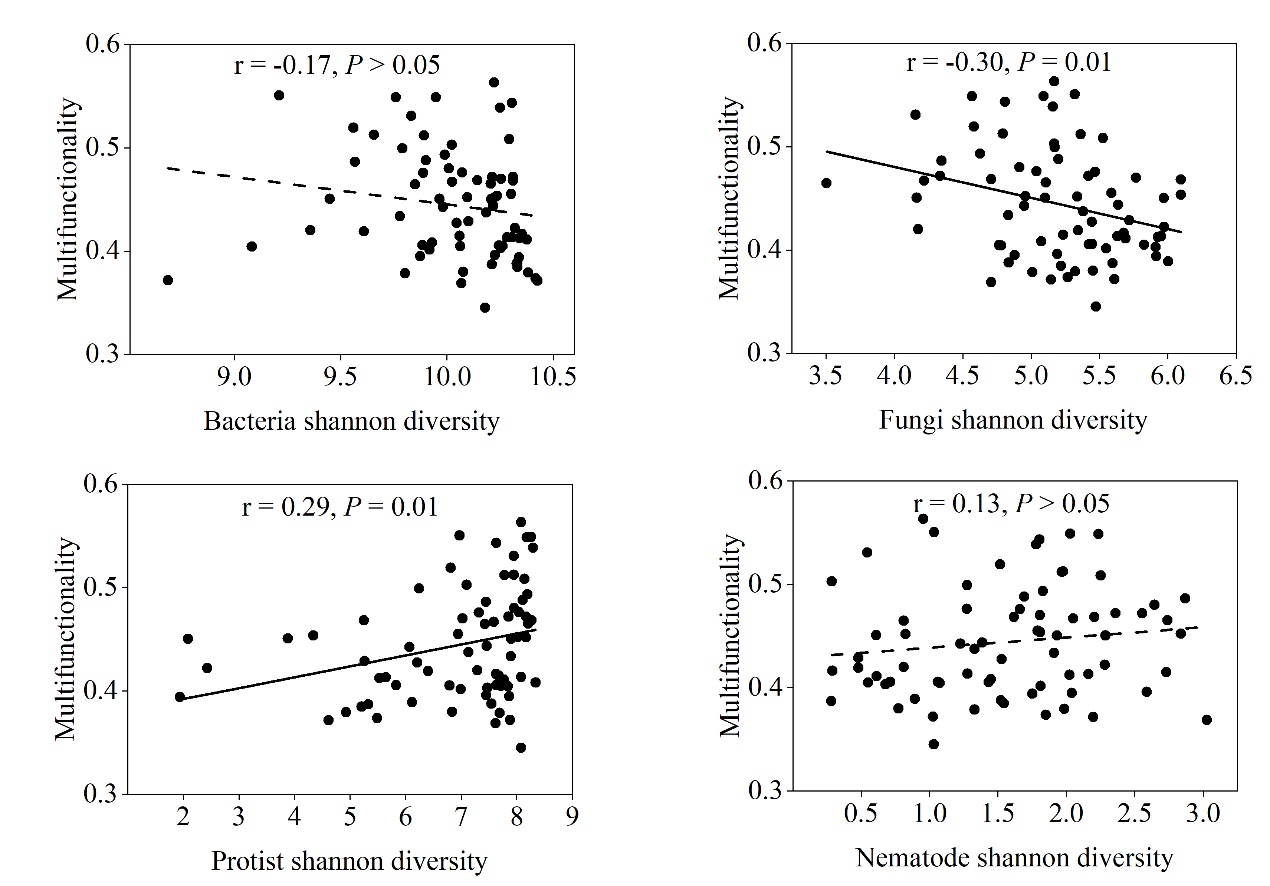


Fig S3. The correlation between microbial Shannon diversity and multifunctionality. The linear relationships between multifunctionality and the biodiversity of selected groups of soil organisms (averaged standardized between 0 and 1). Statistical analysis was performed using ordinary least squares linear regressions; P values were indicated by asterisks: *P < 0.05, **P < 0.01, ***P < 0.001, and ****P < 0.0001.


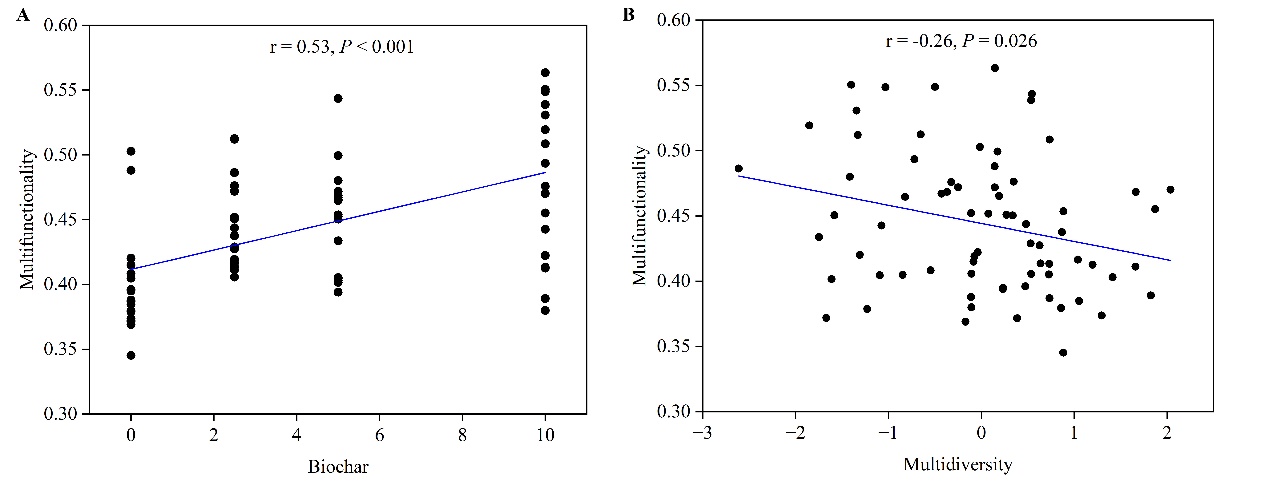


Fig. S4 The relationship between biochar, multidiversity, and multifunctionality. The multidiversity is based on the first axis of the principal coordinate index of bacteria, fungi, protists, and nematodes. Statistical analysis was performed using ordinary least squares linear regressions; P values were indicated by asterisks: *P < 0.05, **P < 0.01, ***P < 0.001, and ****P < 0.0001.
